# Obituary

**Published:** 1882-11

**Authors:** 


### Obituary.

Dr. Oscar Eugene Wilson was born at Chambersburg, Pike county, Illinois, March 24, 1858; died at Versailles, Brown county, Illinois, October 24, 1882, aged 24 years 7 months.

The subject of this notice in his boyhood manifested a very studious disposition, and at the early age of 17 began teaching school. He taught six terms of school, and in 1879 entered the *College of Physicians and Surgeons* at Keokuk, Iowa; graduated in medicine in 1881, and entered into practice with his father, Dr. B. Wilson.

In the practice of his profession he had already proven himself to be worthy of the confidence of the people; as a student he was unexcelled; as a citizen he was above reproach.

About the first of June, 1882, he was attacked with suppurative phlebitis, and was confined to the house for a few days. He then continued his practice until October 5th. At this time his condition became dangerous. His father called in medical assistants who did all for him that was possible to do; one or more physicians were with him all the time, day and night, but all to no purpose; he breathed his last, suddenly, about four o'clock, P. M., surrounded by the stricken family and friends and supported by his aged father.

In the death of Dr. Wilson the medical profession of Brown county has lost one of its most studious, energetic and temperate members, the Masonic fraternity one of their most worthy members; the village of Versailles one of its best citizens. His parents have lost a dutiful and worthy boy, and his brother and sisters a faithful counselor.

After religious services at the house, on Thursday, October 26, the Masonic order took charge of the body and conveyed it to the cemetery at Chambersburg, followed by more than a hundred carriages.

Thus a noble earthly life has ended; but we trust that he has entered into that new life above, where sickness and death can never come, but where the redeemed will ever enjoy the smiles of a loving Saviour. * * *

